# Detection and Analysis of Autoantigens Targeted by Autoantibodies in Immunorelated Pancytopenia

**DOI:** 10.1155/2013/297678

**Published:** 2013-01-31

**Authors:** Hui Liu, Rong Fu, Yihao Wang, Hong Liu, Lijuan Li, Honglei Wang, Jin Chen, Hong Yu, Zonghong Shao

**Affiliations:** Department of Hematology, Tianjin Medical University General Hospital, 154 Anshan Street, Heping District, Tianjin 300052, China

## Abstract

Previously, we described a group of patients with hemocytopenia who did not conform to diagnostic criteria of known hematological and nonhematological diseases. Most patients responded well to adrenocortical hormone and/or high-dose intravenous immunoglobulin treatment, indicating that cytopenia might be mediated by autoantibodies. Autoantibodies were detected on the membrane of various bone marrow (BM) hemopoietic cells by bone marrow mononuclear-cell-Coombs test or flow cytometric analysis. Thus, the hemocytopenia was termed “Immunorelated Pancytopenia” (IRP) to distinguish it from other pancytopenias. Autoantigens in IRP were investigated by membrane protein extraction from BM hemopoietic cells and BM supernatant from IRP patients. Autoantibody IgG was detected in the BM supernatant of 75% of patients (15/20), which was significantly higher than that in aplastic anemia, myelodysplastic syndrome, or autoimmune hemolytic anemia patients (0%) and normal healthy controls (0%) (*P* < 0.01). Autoantigens had approximate molecular weights of 25, 30, 47.5, 60, 65, 70, and 80 kDa, some of which were further identified by mass fingerprinting. This study identified that a G-protein-coupled receptor 156 variant and chain P, a crystal structure of the cytoplasmic domain of human erythrocyte band-3 protein, were autoantigens in IRP. Further studies are needed to confirm the antigenicity of these autoantigens.

## 1. hIntroduction

Over the last decade, we have described a group of patients with hemocytopenia who did not conform to the diagnostic criteria of known hematological and nonhematological diseases, such as aplastic anemia (AA), myelodysplastic syndrome (MDS), paroxysmal nocturnal hemoglobinuria (PNH), megaloblastic anemia (MA), iron deficiency anemia (IDA), anemia of chronic disease (ACD), autoimmune hemolytic anemia (AIHA), or congenital anemia. Anemia, bleeding, and infection are the main manifestations of this hemocytopenia. Most patients had a good response to adrenocortical hormone (ACH) and/or high-dose intravenous immunoglobulin (IVIG) treatment, which indicated that the cytopenia might be mediated by autoantibodies [1–3]. We detected autoantibodies on the membrane of BM hemopoietic cells by bone marrow mononuclear-cell-(BMMNC-) Coombs test [4–6] or flow cytometric analysis [7]. The positive rate was 67% and 86%, respectively [7], indicating that this was an autoimmune disease. We termed this abnormality “Immunorelated Pancytopenia” (IRP). An in-depth study of its pathogenic mechanisms [2, 3] indicated that autoantibodies could inhibit or destroy hemopoietic cells by activating macrophages [8] or complement factors [9] and blocking functional antigens [10]. The production of autoantibodies in this disease may be due to abnormal numbers and altered functions of B lymphocytes [11], caused by inhibition of regulatory T cells (Treg) [12], T helper (Th) 1, and activated Th2 [13] and Th17 [14] cells. Differentiating IRP from other diseases was beneficial not only for the treatment of these patients but also for treating other bone marrow abnormalities, such as AA, MDS, and AIHA [15, 16].

However, the identity of autoantigens in IRP is not known. The identification of autoantigens in autoimmune diseases, such as systemic lupus erythematosus [17], severe asthma [18], and allergic rhinitis [19], helped develop targeted therapies. Our study tried to identify IRP-related autoantigens on the membrane of bone marrow (BM) cells by proteomics.

## 2. Materials and Methods

### 2.1. Patients

All patients were diagnosed as IRP according to the following features [1]: (1) hemocytopenia or pancytopenia with normal or higher percentages of reticulocyte and/or neutrophils; (2) BM: normal or higher percentage of erythroid cells, erythroblastic islands are easy to see; (3) exclusion of other primary and second hemocytopenia disorders; (4) BMMNC-Coombs test (+) or/and autoantibodies on the membrane of BM hemopoietic cells (+) tested by flow cytometry (FCM). Twenty untreated patients (11 males, nine females) were enrolled in our study with a median age of 29 years (range 14–43 years). All patients were inpatients of Tianjin Medical University General Hospital from February to July 2009. Ten mL samples were taken from their ilia. Thirteen controls (5 AA, 5 MDS, and 3 AIHA) were inpatients of our hospital and were diagnosed according to the international criteria of AA, MDS, and AIHA. Ten normal controls from thoracic surgery were also enrolled in this study. BM samples were taken from their postoperative discarded ribs.

### 2.2. BMMNC-Coombs Test

BM mononuclear cells rather than peripheral red cells were used to perform the Coombs test [20]. Fresh heparinized BM samples (5 mL) were diluted with phosphate buffered saline (PBS) in a 1 : 1 proportion, layered over the lymphocyte separation medium and centrifuged at a low speed for 20 min. During centrifugation, differential migration resulted in the formation of several cell layers. Because of their density, lymphocytes and other mononuclear cells were found at the plasma-lymphocyte separation medium interface. Cells were recovered by aspirating the layer and washing with PBS three times. Cell suspensions of 4-5 × 10^6^/mL in PBS were prepared for testing. Anti-human serums (IgG, IgA, IgM, and C3) were diluted as a working solution. Working solutions were mixed with cell suspensions in a 1 : 1 proportion, and cultured at 37°C for 30 min. Finally, we observed agglutination by microscopy.

### 2.3. FCM Analysis

Fresh heparinized BM samples (400 *μ*L) were washed with PBS three times, then separated into four tubes and stained with either mouse IgG1-FITC, mouse IgG1-PE, mouse IgG1-APC as negative control, or stained separately with CD15-FITC, GlyCoA-FITC, and CD34-FITC (BD Pharmingen, San Diego, CA, USA). Anti-human IgG-PE and anti-human IgM-APC (BD PharMingen) were added to each tube. After incubation in the dark for 30 min at 4°C, cells were incubated with 2 mL erythrocyte lytic solution (BD PharMingen) for 10 min at room temperature and washed three times with PBS. Finally, at least 30 000–100 000 cells were acquired and analyzed on a FACSCalibur flow cytometer (BD Biosciences).

### 2.4. Membrane Protein Extraction

Hemopoietic cells were separated from BM of patients with IRP and healthy volunteers by erythrocyte lytic solution. Membrane proteins were extracted by Mem-PER Eukaryotic Membrane Protein Extraction Reagent Kit (Thermo Scientific Pierce, Rockford, IL, USA). Protein concentration was determined using BCA Protein Assay Kit (Thermo Scientific Pierce). BM supernatant was stored at −80°C until use.

### 2.5. SDS-PAGE and Western Blot

Solubilized proteins were separated using 10% sodium dodecyl sulfate-(SDS-) polyacrylamide gel electrophoresis (PAGE). IgG autoantibodies to BM hemopoietic cells were detected in BM supernatant by western blot. After electrophoresis, the SDS-PAGE gels were stained with Coomassie Brilliant Blue (CBB) or electrotransferred to polyvinylidene difluoride membranes (PVDF) (Bio-Rad Laboratories, Hercules, CA, USA). For PVDF membranes, blocking was performed using 5% nonfat milk at 4°C overnight. Patients' BM supernatant at 1/100 dilution with 5% nonfat milk was applied for 2 h at room temperature. After washing with Tris Buffered Saline, with Tween-20 (TBST), goat anti-human IgG peroxidase conjugate diluted 1/5000 in 5% non-fat milk was applied for 1 h, and electrochemiluminescence (ECL) reagent was used.

### 2.6. In-Gel Digestion, Mass Determination, and Mass Spectrometry

Protein bands on the gel were stained with CBB, which corresponded to the positive bands on the WB membranes. The recovered gel fragments were digested and analyzed by liquid chromatography-mass spectrometry/mass spectrometry (LC-MS/MS) at the Academy of Military Medical Sciences. A list of the peptide masses was compiled using the Mascot software program, in which the NCBI protein databases were searched.

### 2.7. Statistical Analysis

Statistical analysis was performed with SPSS 16.0 statistical software and tested by TANOVA.

## 3. Results

### 3.1. Detection of Autoantibodies to BM Hemopoietic Cells

**Figure 1:**
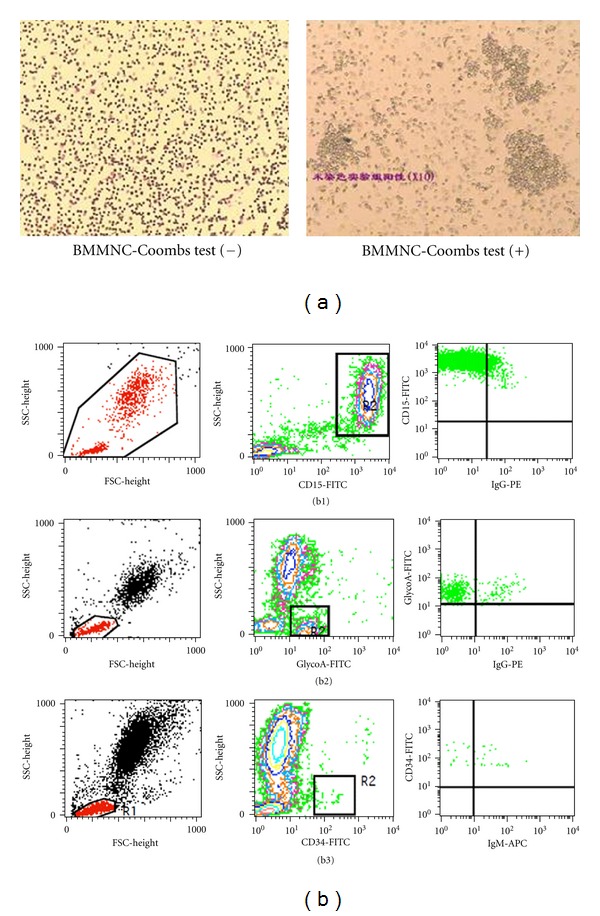
Autoantibodies were detected in IRP patients. (a) BMMNC-Coombs test, (b) flow cytometry analysis. (b1) autoantibodies were detected on granulocytes. (b2) autoantibodies were detected on nucleated erythrocytes. (b3) autoantibodies were detected on stem cells.

We first tried to understand the overall profiles of autoimmunity in patients with BMMNC-Coombs test positive hemocytopenia. To achieve this, we detected autoantibodies/autoantigens by means of SDS-PAGE and subsequent WB using 43 BM supernatant samples from 20 patients with IRP (Table 1, Figure 1), five patients with AA, five patients with MDS, three patients with AIHA, and 10 normal controls. Proteins extracted from BM hemopoietic cells were separated by SDS-PAGE. Next, the separated proteins were transferred onto membranes and then were reacted with each of the 43 BM supernatant samples by western blot. Autoantibody IgG targeting BM cells were observed in BM supernatant of IRP patients at a positive rate of 75% (15/20), which was significantly higher than for AA, MDS, or AIHA patients (0%) and normal controls (0%) (*P* < 0.01).

### 3.2. Identification of 30 kd BM Hemopoietic Cells Autoantigens

**Figure 2:**
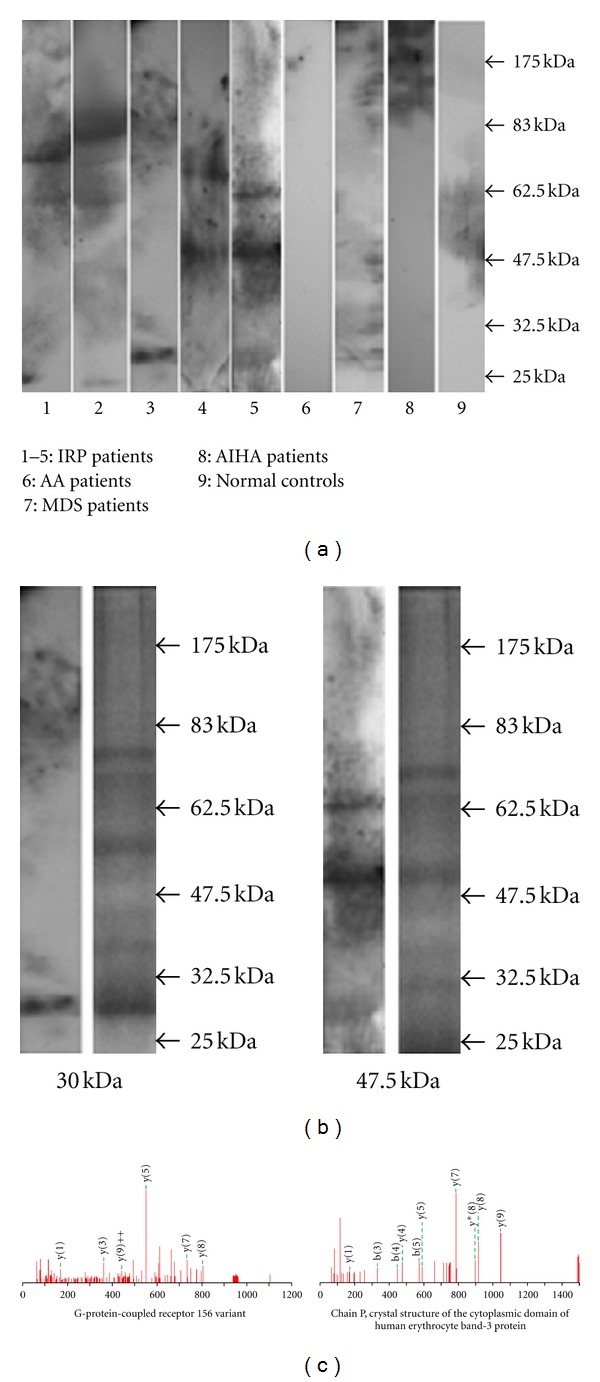
Autoantigens targeted by IgG autoantibody isolated and identified by SDS-PAGE, western blot, and LC-MS/MS. (a) Autoantibody IgG in patients reacted with several autoantigens with approximate MWs of 25, 30, 47.5, 60, 65, 70, and 80 kDa. (b) 30 and 47.5 kDa were identified by LC-MS/MS (L: WB, R: gels stained with CCB). (c) Results of MS.

Seven major protein bands reactive to at least one of the samples from the patients with IRP were observed. The approximate molecular weights (MWs) of the seven major protein bands were 25, 30, 47.5, 60, 65, 70, and 80 kDa (Figure 2), and the positive rates were 30% (6/20), 20% (4/20), 15% (3/20), 10% (2/20), 15% (3/20), 10% (2/20), and 5% (1/20), respectively (Table 2). The detection of multiple autoantigens indicated that autoimmunity directed toward various self-proteins may be a common phenomenon in these patients.

We next tried to identify the two major protein bands by LC-MS/MS. Specifically, peptides extracted from the corresponding gel bands after digestion with trypsin were subjected to mass measurement and computer searching. We successfully identified the 30 kDa protein band as two autoantigens, a G-protein-coupled receptor 156 variant, and chain P, crystal structure of the cytoplasmic domain of human erythrocyte band-3 protein (Figure 2).

## 4. Discussion

IRP is a disease independent from other pancytopenic diseases described in recent years. Infection, anaphylaxis, and pregnancy are suspected risk factors associated with this patient group. A number of patients (72.5%, 145/200) had pancytopenia. The median percentage of reticulocytes was 1.8%. More than half the patients had hyper-BM cellularity with a higher percentage of nucleated erythroid cells in the sternum [21]. Autoantibodies on BM hemopoietic cells were detected by BMMNC-Coombs test and/or flow cytometry. An in-depth study of its pathogenic mechanism showed that IRP is an autoimmune disease where autoantibodies target BM hemopoietic cells. A study in SLE by Fu et al. [22] showed that more than 50% of patients with SLE were observed byBMMNC-Coombs test positive, approximately 60% of which had blood cytopenia. These results indicate that IRP was an autoimmune disease, similar to SLE, but where the target organ was BM.

However, the autoantigens in IRP are unknown. Therefore, we used a proteomic approach, a combination of SDS-PAGE and subsequent mass spectrometry [23]. By screening SDS-PAGE-WB using BM supernatant samples from patients with IRP, AA, MDS, AIHA, and normal controls, we found seven major protein bands in IRP but not in AA, MDS, AIHA, and normal controls. These results also indicated that IRP was different from other known hematological diseases, such as AA, MDS, and AIHA. We often found 3-4 protein bands in individual patients, indicating that autoimmunity is a common phenomenon.

Finally, we identified two autoantigens, G-protein-coupled receptor 156 variant and chain P, crystal structure of the cytoplasmic domain of human erythrocyte band-3 protein by LC-MS/MS in IRP [24]. G-protein-coupled receptor 156 variant belongs to the family of G-protein-coupled receptors (GPCRs) [25–27]. Currently, the structure and function of G-protein-coupled receptor 156 are unknown. However, mutations of GPCRs can cause various kinds of diseases [28]. The activation or suppression of GPCRs are important in nerve conduction and are associated with neurogenic disease, heart disease, metabolic disturbance, and cancer. Furthermore, some types of GPCRs can inhibit GTPase and, thus, exhibit negative intrinsic activity, and its affinity for receptors is increased following uncoupling from G proteins [29]. Therefore, GPCR 156 variant may play a role in the suppression of proliferation and differentiation of BM hemopoietic cells in BMMNC-Coombs test positive hemocytopenia by preventing binding with ligands due to structural changes, which could inhibit G protein activity. Alternatively, it could induce continuous signal transduction in the absence of ligand binding due to structural changes. The mechanism of GPCR156 variant in this disease requires further study.

Band-3 protein is important for the membrane stability of red blood cells. Abnormalities of band-3 protein are associated with disease, such as hereditary spherocytosis and hereditary erythroblastic multinuclearity with a positive acidified serum test (HAMPAS) [30]. In our study, autoantibodies should not bind to the cytoplasmic domain of normal nucleated erythrocytes, but in some pathological conditions (oxidative damage, acute/chronic virus infection, and increased calcium concentration), the membrane of cells can be damaged, causing deformity. Changes in the membrane of cardiomyocytes in *Helicobacter pylori* (HP) infection have been observed [31]. Guo et al. [32] reported that anti-HP antibodies could have an effect on chain S, the crystal structure of the cytoplasmic domain of human erythrocyte band-3 protein, which causes damage to red blood cells in gastrointestinal ulcers.

## 5. Conclusions

In conclusion, IRP is a bone marrow abnormality different from other known hemopoietic diseases. Here, we applied a proteomic approach to survey autoantigens and identified two autoantigens. Further studies to confirm the antigenicity of these autoantigens, including the use of a recombinant protein and measuring the prevalence of autoantibodies in IRP, are required.

## Figures and Tables

**Table 1:** Profiles of patients with IRP enrolled in this study.

Pt. no.	Age	Sex	BMMNC-Coombs test	FCM analysis
1	42	F	—	CD15+ cells IgG(+)
2	26	M	IgG(+)	CD15+ cells IgG(+), GlyCoA+ cells IgM(+), CD34+ cells IgM(+)
3	34	F	IgM(+)	GlyCoA+ cells IgM(+)
4	40	F	C3(+)	CD34+ cells IgG(+), IgM(+)
5	32	F	IgG(+), C3(+)	CD15+ cells IgG(+), CD34+ cells IgG(+), IgM(+)
6	30	M	—	GlyCoA+ cells IgM(+)
7	23	M	IgM(+)	GlyCoA+ cells IgG(+), IgM(+)
8	27	M	—	CD15+ cells IgM(+)
9	22	M	—	CD34+ cells IgM(+)
10	25	F	—	CD34+ cells IgG(+), IgM(+)
11	14	M	IgM(+)	GlyCoA+ cells IgM(+)
12	14	M	IgM(+)	GlyCoA+ cells IgG(+), IgM(+), CD34+ cells IgM(+)
13	19	M	IgM(+)	CD15+ cells IgM(+)
14	36	F	—	CD34+ cells IgG(+), IgM(+)
15	30	F	IgM(+)	GlyCoA+ cells IgM(+)
16	29	M	IgM(+)	CD15+ cells IgM(+), CD34+ cells IgG(+), IgM(+)
17	30	M	—	CD34+ cells IgM(+)
18	26	M	IgM(+)	CD15+ cells IgM(+), GlyCoA+ cells IgM(+)
19	29	M	—	CD34+ cells IgG(+), IgM(+)
20	43	F	IgG(+)	CD15+ cells IgG(+), IgM(+)

Pt. no.: patient number; Ig: immunoglobulin; FCM: flow cytometry; GlyCoA; glycine coenzyme A; BMMNC: bone marrow mononuclear cell; F: female; M: male.

**Table 2:** Protein bands reactive with BM supernatant samples from IRP patients.

Pt. no.	Protein bands						
	25 kDa	30 kDa	47.5 kDa	60 kDa	62.5 kDa	70 kDa	80 kDa
1						+	
2				+		+	+
3				+			
4		+					
5			+		+		
6	+						
7			+		+		
8	+	+					
9	+						
10	+						
11							
12		+					
13	+						
14							
15							
16			+		+		
17	+						
18		+					
19							
20							

Pt. no.: patient number.
